# Clinical helminth infections alter host gut and saliva microbiota

**DOI:** 10.1371/journal.pntd.0010491

**Published:** 2022-06-08

**Authors:** Geoffrey N. Gobert, Louise E. Atkinson, Andrea Lokko, Tippayarat Yoonuan, Orawan Phuphisut, Akkarin Poodeepiyasawat, Nirundorn Homsuwan, Angela Mousley, Poom Adisakwattana

**Affiliations:** 1 School of Biological Sciences, Queen’s University Belfast, Belfast, United Kingdom; 2 Department of Helminthology, Faculty of Tropical Medicine, Mahidol University, Bangkok Thailand; Texas Biomedical Research institute, UNITED STATES

## Abstract

**Background:**

Previous reports show altered gut bacterial profiles are associated with helminth infected individuals. Our recently published molecular survey of clinical helminthiases in Thailand border regions demonstrated a more comprehensive picture of infection prevalence when Kato Katz microscopy and copro-qPCR diagnostics were combined. We revealed that *Opisthorchis viverrini*, hookworm, *Ascaris lumbricoides* and *Trichuris trichiura* were the most predominant helminth infections in these regions. In the current study, we have profiled the faecal and saliva microbiota of a subset of these helminth infected participants, in order to determine if microbial changes are associated with parasite infection.

**Methods:**

A subset of 66 faecal samples from Adisakwattana et al., (2020) were characterised for bacterial diversity using 16S rRNA gene profiling. Of these samples a subset of 24 participant matched saliva samples were also profiled for microbiota diversity. Sequence data were compiled, OTUs assigned, and diversity and abundance analysed using the statistical software Calypso.

**Results:**

The data reported here indicate that helminth infections impact on both the host gut and oral microbiota. The profiles of faecal and saliva samples, irrespective of the infection status, were considerably different from each other, with more alpha diversity associated with saliva (*p-value*≤ 0.0015). Helminth infection influenced the faecal microbiota with respect to specific taxa, but not overall microbial alpha diversity. Conversely, helminth infection was associated with increased saliva microbiota alpha diversity (Chao 1 diversity indices) at both the genus (*p-value* = 0.042) and phylum (*p-value* = 0.026) taxa levels, compared to uninfected individuals. Elevated individual taxa in infected individuals saliva were noted at the genus and family levels. Since *Opisthorchis viverrini* infections as a prominent health concern to Thailand, this pathogen was examined separately to other helminths infections present. Individuals with an *O*. *viverrini* mono-infection displayed both increases and decreases in genera present in their faecal microbiota, while increases in three families and one order were also observed in these samples.

**Discussion:**

In this study, helminth infections appear to alter the abundance of specific faecal bacterial taxa, but do not impact on overall bacterial alpha or beta diversity. In addition, the faecal microbiota of *O*. *viverrini* only infected individuals differed from that of other helminth single and dual infections. Saliva microbiota analyses of individuals harbouring active helminth infections presented increased levels of both bacterial alpha diversity and abundance of individual taxa. Our data demonstrate that microbial change is associated with helminthiases in endemic regions of Thailand, and that this is reflected in both faecal and saliva microbiota. To our knowledge, this is the first report of an altered saliva microbiota in helminth infected individuals. This work may provide new avenues for improved diagnostics; and an enhanced understanding of both helminth infection pathology and the interplay between helminths, bacteria and their host.

## Author summary

In both humans and animals, microbiomes are known to influence general health. Recently, several human studies demonstrated that intestinal helminth infections enhanced both the abundance and diversity of gut microbiomes, which may prevent the host from developing inflammatory diseases. Gastrointestinal helminth infections are considered to predominantly impact on the local microbiome of the host gut. Microbiome remodelling in other, more distant, parts of the body as a result of helminth infection has not been investigated. Here we characterized the alteration of host gut microbiota during infection with major gastrointestinal helminth species including, *O*. *viverrini*, hookworm, *A*. *lumbricoides* and *T*. *trichiura* in rural populations of Thailand. The data indicate that helminth infections, including *O*. *viverrini*, altered the abundance of specific bacterial taxa in the host gut but not overall bacterial diversity. In contrast, the saliva microbiota displayed both altered bacterial richness and abundance during active helminth infections. To our knowledge, these data provide the first evidence that intestinal helminth infections may alter not only the host gut microbiota, but also influence microbial communities in other, more peripheral, regions including the saliva.

## Introduction

Parasitic helminth infections are a global health burden, especially to people living in economically underdeveloped areas where poor sanitation levels and malnutrition increase disease transmission potential. Approximately two billion people are at risk of infection by gastrointestinal helminths [1], which can be contracted by accidental ingestion (food borne or soil transmitted) [2]. Soil transmitted helminths (STHs) account for approximately 3.3 million DALYs lost globally each year [3]. Southeast Asia carries the STH infection burden where almost one-third of global helminthiases are recorded [4]. *Strongyloides stercoralis*, *Opisthorchis viverrini*, hookworm, *Ascaris* and *Trichuris* are major endemic species in Northern Thailand [5,6]. Many of these helminth infections have a systemic impact on human health via interactions with the host immune response [7].

In humans multiple organs and tissues, for example the gastrointestinal tract, contain rich microbial communities. Since gastrointestinal helminths and microbes share the same host environmental location within the host gut, it is proposed that co-evolution of these pathogens [8] exerts influence on the composition of the gut microbiota [9]. Evidence of the impact helminths have on the host microbiota has been derived from animal models and clinical studies. However the data between and within studies can be highly variable and no clear consensus is evident [10]. Human studies are particularly inconsistent with respect to correlations between the gut microbiota and helminth infections. Whilst some clinical studies indicate that helminth infections are associated with increased microbiota alpha diversity in the gut [11,12], other reports have indicated only a marginal impact [13]. The influence of soil-transmitted helminths including *Ascaris lumbricoides* on the human gut microbiota has been reported from endemic regions [14], where *Lachnospiracae* was reduced and twelve other bacterial taxa were increased in individuals with active *A*. *lumbricoides* infection.

Clearer microbiota shifts have been identified in animal models. This approach allows mitigation of many of the variable factors associated with human studies including diet, geographical location, the duration of helminth infection, and the presence of co-morbidities. Wu *et al*. (2008) studied the dynamics of proximal colon microbiota in pigs during *Trichuris suis* infection [15], reporting increases in populations of *Campylobacter spp*., *Paraprevotella* and *Micispirillum* in infected animals. In mouse studies, *Lactobacillus* increased in animals harbouring *Heligmosomoides polygyrus* [16].

While studies of the gut microbiota have been a major focus of disease associated microbial dysbiosis, more recently interest in the saliva microbiota as an indicator of oral and general health has increased [17]. The saliva represents a particularly non-invasive approach to identifying microbial changes reflective of the presence of disease. Microbiota changes have been identified in the saliva of patients with systemic conditions including HIV, diabetes, cancer, and rheumatoid arthritis [17]. In addition, several reports have correlated alterations in the oral microbiota with changes in the faecal microbiota [18]. Due to the directional transfer of microbiota from the oral cavity to the gut, researchers have also sought to associate digestive tract cancers with changes in the salivary microbiota [19]. While oral bacteria can colonise the gut [20], it is not known if the gut bacteria, including pathogenic organisms, can also populate the oral microbiota. Collectively, these observations demonstrate the potential of saliva as a less invasive alternative to faecal sampling for microbiota profiling of systemic or distal disease states including helminth infections.

Whilst there is significant interest in exploiting microbiome profiles as diagnostic tools to detect diseases that induce bacterial dysbiosis [21], conflicting reports associating disease to altered microbiomes make bacterial biomarkers difficult to identify. Encouragingly, larger meta-analysis studies have provided some more consistent data relating to microbiome changes for diseases such as colorectal cancer [22]. Establishing a microbiome signature for specific disease states will be the first step in the development of an effective diagnostic assay [21]. Despite the difficulties associated with microbial profiling against the backdrop of disease, microbiome diagnostics for use in clinical practice remains an exciting and potentially powerful tool for the identification and targeted treatment of a wide range of morbidities, including helminth disease [23].

In a recent study, we demonstrated more accurate helminth infection prevalence data in a Southeast Asia cohort study using molecular diagnostics [6]. Here, using this diagnostic information, we present the impact that active helminth infections have on the host faecal and saliva microbiomes. To our knowledge, human oral (saliva) microbiota signatures, in conjunction with active helminth infection, has not previously been reported.

## Methods

### Ethics statement

This study, which included the original collection of human stool and saliva samples and their examination using microscopy and molecular analysis, was approved by the Human Research Ethics Committee of the Faculty of Tropical Medicine, Mahidol University, Bangkok, Thailand (MUTM 2021-042-01). Informed verbal consent was obtained from all individuals.

### Collection of samples and DNA extraction

The original collection of faecal samples from individuals and the subsequent molecular and microscopic characterisation of active helminth infection was reported in Adisakwattana *et al*., [6]. Participants in this original study were recruited without the presentation of symptoms. Briefly, faecal samples were provided by 567 study participants from three Thai border sites including villages within the Tak Province close to the Thai-Myanmar border; Ubon Ratchathani Province at the Thai-Lao border; and Sisaket Province at close proximity to the Thai-Cambodia border, and in field Kato Katz microscopy was performed to confirm helminth infection status [6]. Saliva samples were also obtained in Ubon Ratchathani Province and matched to faecal samples from the same individuals collected concurrently. Faecal and saliva samples were stored in 80% (v/v) ethanol for transport at ambient temperature to Bangkok for molecular extraction and analyses. DNA from both faeces and saliva was isolated using QIAamp Fast DNA Stool Mini Kit (Qiagen GmbH, Hilden, Germany), and stored at -80°C.

### Microbiota profiling

**Fig 1:**
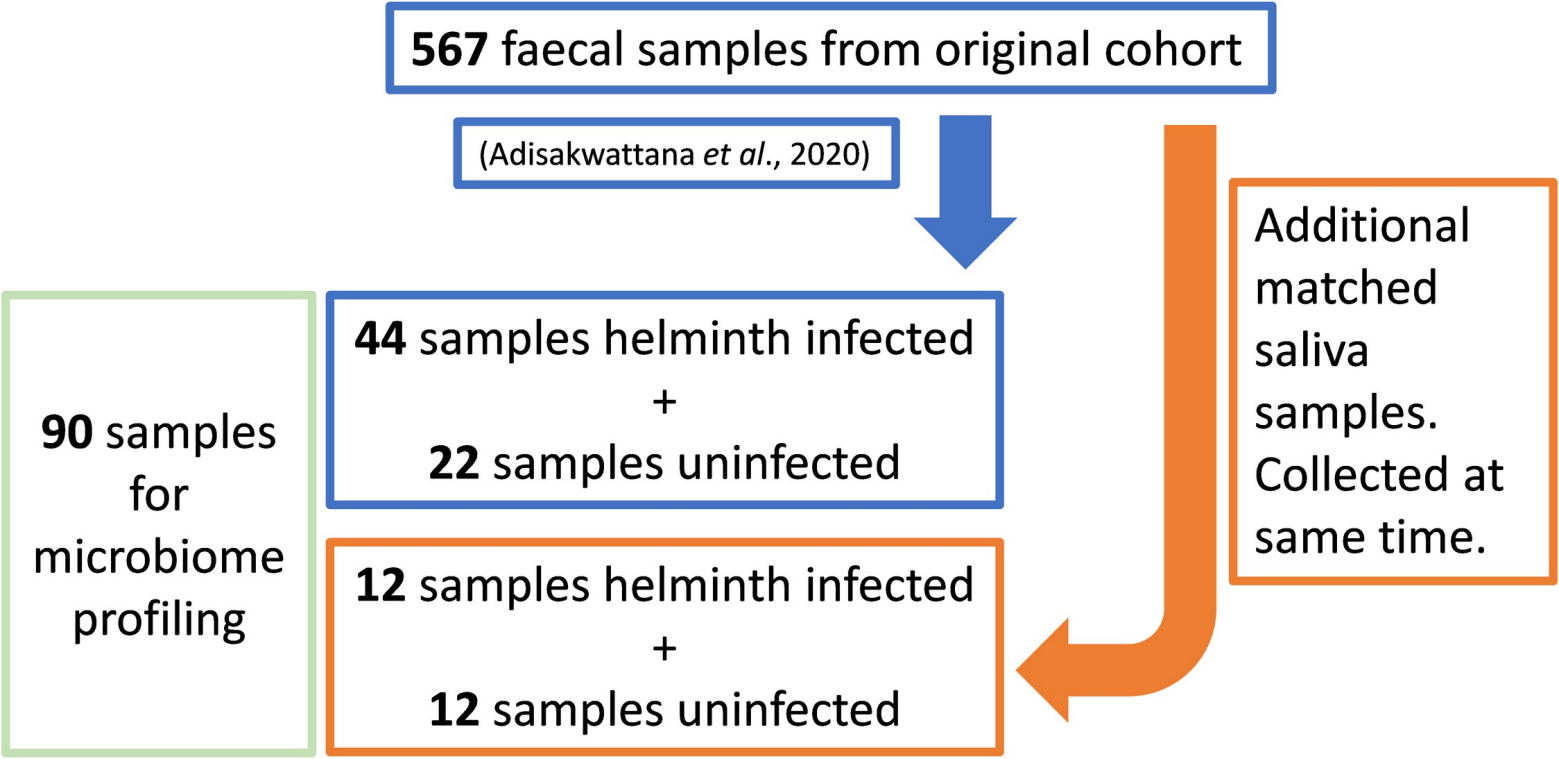
Study design of samples selected for microbiota profiling. From the original cohort [6], a subset of faecal samples was selected and of these a further subset of matched saliva samples, were used for 16S profiling.

A total of 90 samples, either faecal (66) or saliva (24), were used for microbiota profiling (**Fig 1**). From the original study [6], a subset of 44 faecal samples from infected individuals and 22 faecal samples from uninfected individuals were selected for microbiota profiling. Patient matched saliva samples obtained from 12 infected and 12 uninfected individuals within the faecal sample cohort (saliva samples were obtained at the same time as faecal collection), were also analysed. A mock kitome control was included.

Microbiota profiles were generated using the 16S Ribosomal RNA Gene Amplicons Workflow (Centre for Genomic Research, University of Liverpool), following their standard protocols. Primers previously described [24] were used to amplify the V4 region of 16s rRNA. PCR products were purified using AMPure SPRI Beads before being quantified using Qubit and assessed using the AATI Fragment Analyzer. Libraries were sequenced on an Illumina MiSeq platform with V2 chemistry using sequencing by synthesis (SBS) technology to generate 2x250 bp paired-end reads.

### Initial read processing and quality assessment

Briefly, base-calling and de-multiplexing of indexed reads was performed by CASAVA version 1.8.2 (Illumina) to produce data files in FASTQ format. The raw FASTQ files were trimmed to remove Illumina adapter sequences using Cutadapt version 1.2.1 [25]. The option “-O 3” was set, so the 3’ end of any reads which matched the adapter sequence over at least 3 bp was trimmed off. The reads were further trimmed to remove low quality bases, using Sickle version 1.200 with a minimum window quality score of 20. After trimming, reads shorter than 20 bp were removed. If both reads from a pair passed this filter, each was included in the R1 (forward reads) or R2 (reverse reads) file. If only one of a read pair passed this filter, it was included in the R0 (unpaired reads) file. Sequences for each sample were merged into a single file was used for the analysis by using a custom pipeline based on QIIME1.9.1 [26]. GreenGenes database (version 13.8) was used throughout the analysis. The analysis resulted in a phylogeny of the OTUs and an OTU table file (in “biom” format), which contains the OTU information for each sample as well as taxonomic information on the OTUs and sample metadata.

### Calypso microbiota profile analysis

Initial analysis of the OTU abundance data normality using D’Agostino-Pearson, and additional follow-up tests for read numbers were performed using GraphPad (9.3.1). Calypso (version 8.54) [27] was used for mining the microbiota dataset and for data visualisation. Rarefied and filtered OTU tables were uploaded to Calypso and square root (SQR) transformed with TSS (total sum normalisation). Samples with less than 1,500 reads were removed (excluding the kitome). Microbial alpha diversity was characterised using the Simpson, Chao1, Shannon and Richness diversity indices. Correlations with continuous variables (such as tissue type or tissue and infection status) were calculated using ANOVA. Significant taxa were identified by ANOVA. With statistical significance considered at *p-value*≤ 0.05, with “not significant” indicated by *NS*. Multivariate data visualization and multivariate statistical testing were used to indicate the presence of multifaceted associations between microbial communities. For beta diversity both supervised Redundancy Analysis (RDA) and Canonical Correspondence Analysis (CCA) and unsupervised Principal Coordinates Analysis (PCoA) methods were used at the OTU level unless noted.

## Results and discussion

### Metadata of selected samples

The helminth infection profiles of the 66 cohort participants selected for this study, based on the original PCR and Kato Katz diagnostics [6], are presented in metadata **S1 Table**. Of the 44 helminth positive individuals, 18 were infected with *O*. *viverrini*; either alone (12 individuals) or as a coinfection with other helminth species (6 individuals). The next most common infection was *A*. *lumbricoides* (10 individuals infected; 3 mono-infection). Ten of the 12 cohort participants selected for saliva sampling had an active *O*. *viverrini* infection, as determined by faecal diagnostics.

### Sequencing quality indicators

Major indicators of the quality of the microbiota profiling are presented in **S1 Fig**. Rarefaction analysis indicated that the metagenomic sequence data generated from the sample reads was representative of the diversity of the microbial communities present to a satisfactory level (**S1A Fig**). The flattening of the species richness curve to the right of the graph indicates that further sampling is unlikely to yield additional species in either microbiota [28]. A summary of the reads per sample are shown as individual samples (**S1B Fig**), and as statistically analysed groups by infection status (**S1C and S1D Fig**). The kitome present the lowest reads at 45 (**S1C Fig**), and faecal and saliva samples ranging between 54,266–200,180 reads. The statistically significant (*p-value* = 0.02329) variation of reads between sample types (excluding kitome control) indicated that TSS normalization was warranted (**S1D Fig** Follow up Tukey’s multiple comparisons test indicated the following groups were significantly different (*p-value*) K vs. FI (0.0001), K vs. FU (<0.0001), SU vs. K (0.0015), SI vs. K (<0.0001) and SU vs. FU (0.0209).

The OTU abundance did not pass a D’Agostino-Pearson normality test (alpha = 0.05). ANOVA analysis was used to compare both infection status and tissue type and was maintained for further analysis. ANOVA while not an optimal test for non-normally distributed data, empirical evidence for its robustness under these conditions, supports its use in this study for consistency [29]. Microbiome data often presents non-normality and the use of multi-variant analysis can be beneficial [30]. It has been reported that 16S rRNA sequencing does not provide enough resolution to distinguish and identify accurately bacterial species. The use of sub-regions, cannot provide the taxonomic accuracy compared to sequencing the full 16S gene [31]. The use of QIIME rather than QIIME2 pipeline also limits the identification of species level differences [32], as reflected in the large number of “unclassified species present. As such we have limited the taxonomic ranks within our analysis, while retaining OTUs and species level analysis in S2–S6 Tables.

### Saliva and faecal samples present different microbiota, with greater bacterial diversity associated with saliva

**Fig 2:**
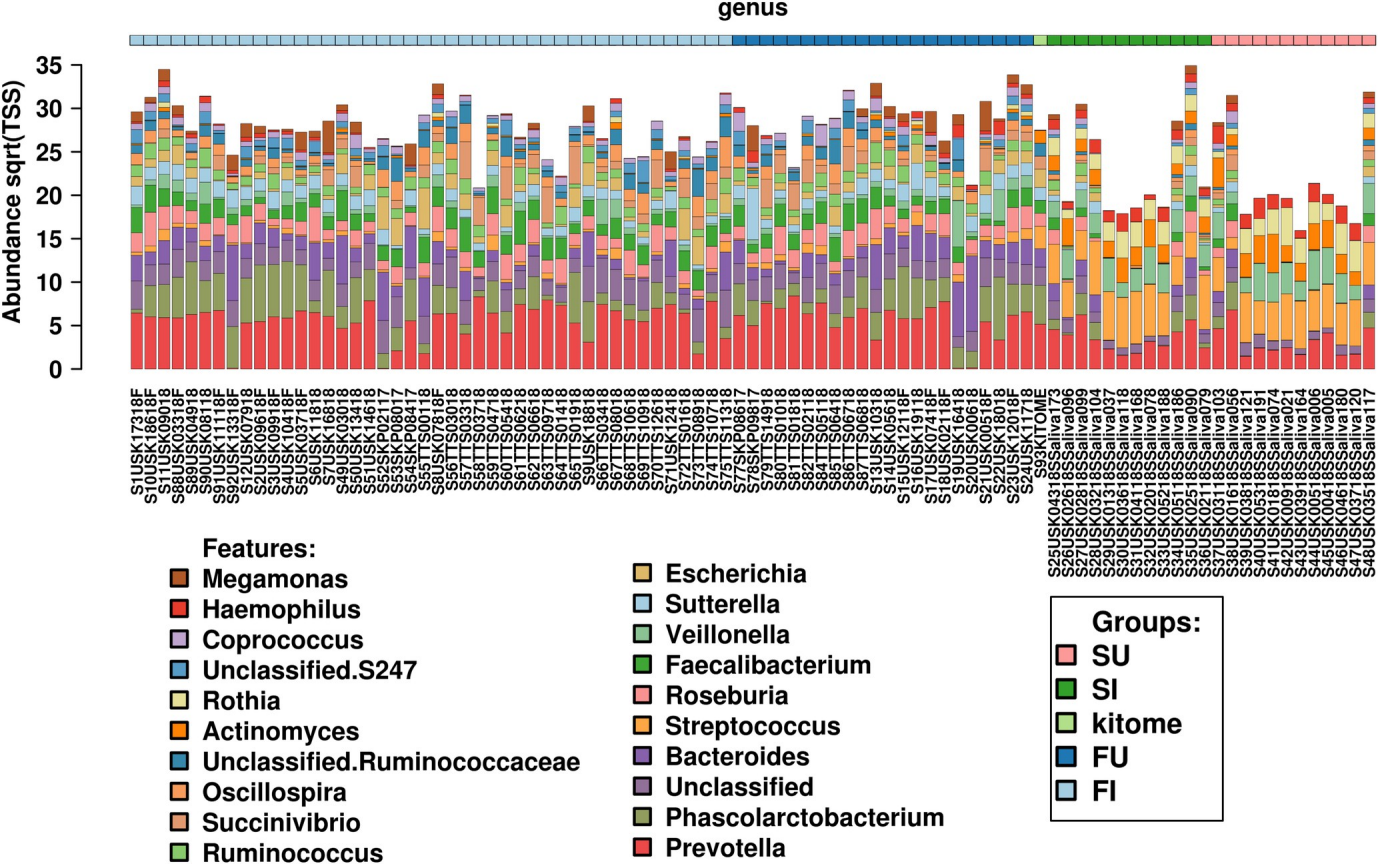
Bacterial populations differences between faecal and saliva samples. Non-clustered bar-chart summary of microbiota profiling of clinical samples and kitome control. Major bacterial genera associated with saliva or faecal samples in infected and uninfected hosts. FU = Faecal Uninfected, FI = Faecal Infected, SU = Saliva Uninfected, SI = Saliva Infected.

**Fig 3:**
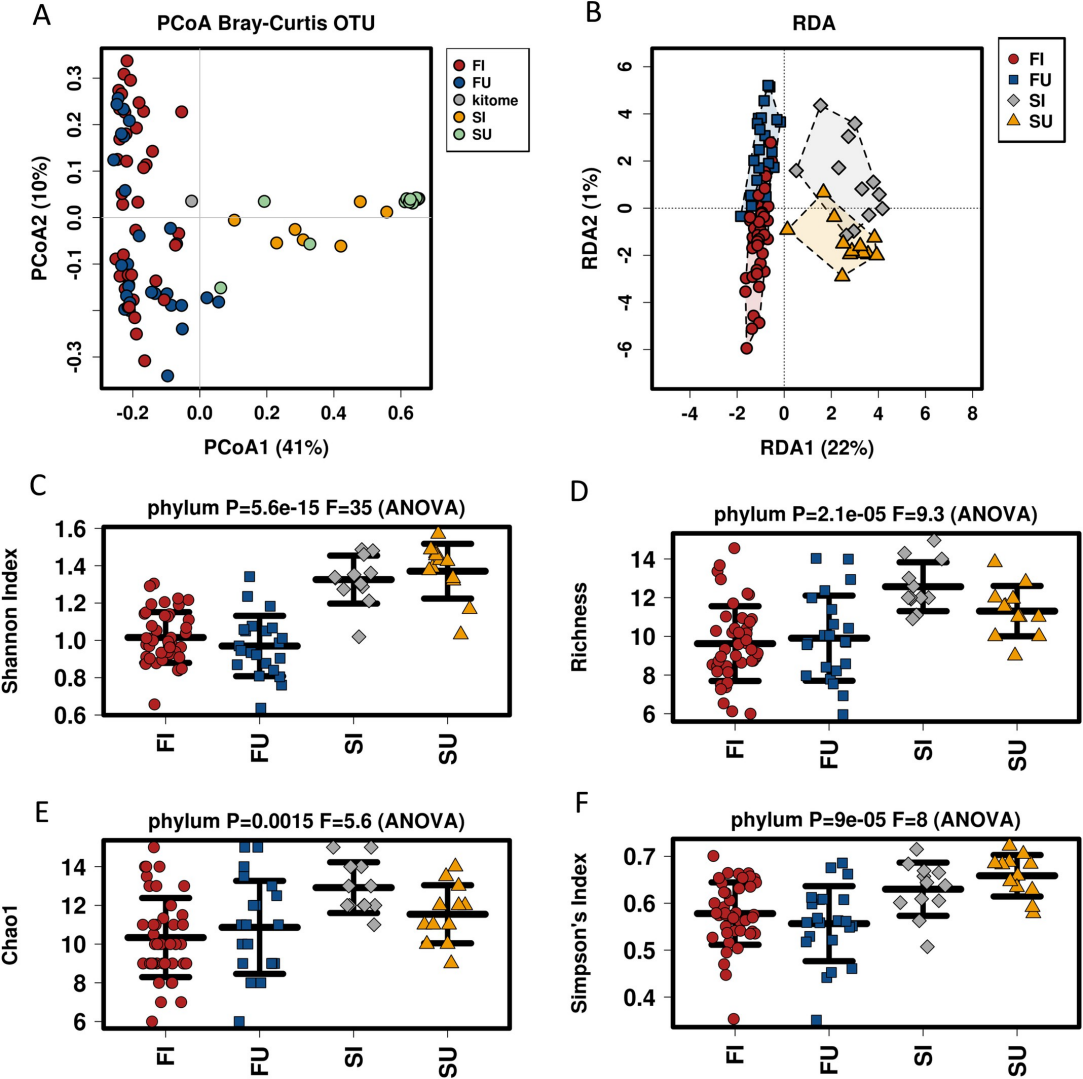
Multivariate and alpha diversity analysis of faecal and saliva sample by infection status, demonstrates differences in the microbiota of these two biological samples. A. Unsupervised Principal Coordinates Analysis (PCoA) methods were used at the OTU level B. Redundancy Analysis (RDA- OTU level) demonstrating that differences between faecal and saliva samples were statistically significant (*p-value* = 0.001). C-F. All four diversity analysis methods indicated statistical differences between the sample sources at the phylum level. FU = Faecal Uninfected (blue), FI = Faecal Infected (red), SU = Saliva Uninfected (yellow), SI = Saliva Infected (grey).

Differences in the bacterial populations between faecal and saliva samples were evident at both the species and genus levels (**Fig 2**), irrespective of infection status. The non-clustered bar-chart shows the most abundant taxa present in the faecal and saliva samples including *Coprococcus*, *Haemophilus*, *Actinomyces*, and *Megamonas* genera. To further investigate these differences multivariate analysis (**Fig 3)**, with supervised RDA was conducted; this showed statistically significant (*p-value* = 0.001) differences in the microbiota between faecal and saliva samples (**Fig 3B**), irrespective of infection status. The alpha diversity of the saliva samples was increased in all four metrics used (Simpson, Chao1, Shannon and Richness diversity indices) independent of infection status. This was evident at the genus levels and is shown at the phylum level (*p-value*≤ 0.0015) in **Fig 3C–3F**.

ANOVA analysis of the four sample types (faecal uninfected, faecal infected, saliva uninfected, saliva infected) is presented in **S2 Table**, with OTU, species, genus, family, order, and phylum stratification. Statistically significant differences were observed between faecal and saliva microbiota, irrespective of infection status (infected/uninfected faeces compared to saliva), presenting 108/108 species, 82/79 genera, 44/41 families, 24/21 orders, and 12/8 phyla.

### Helminth infections alter the abundance of specific bacterial taxa in the gut

**Fig 4:**
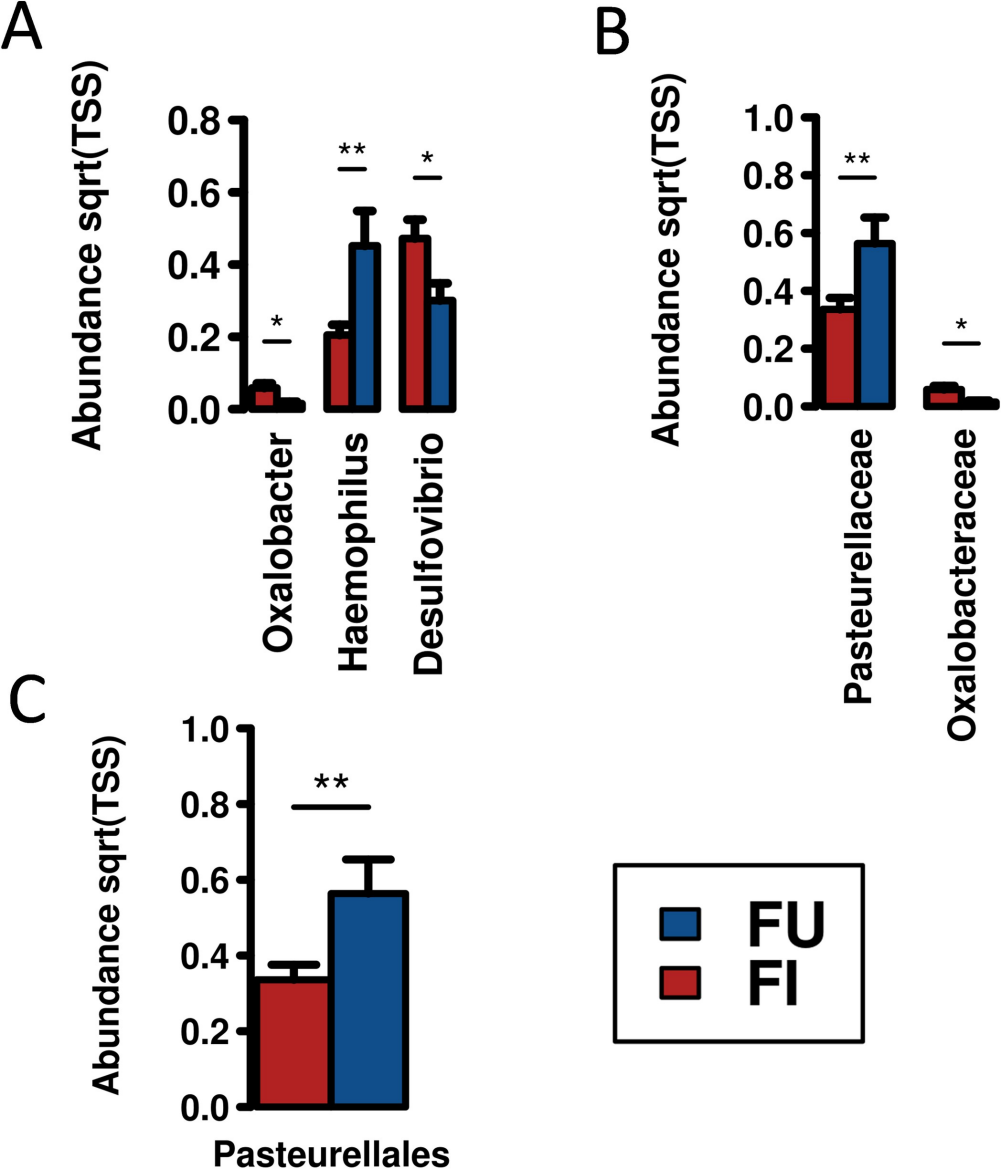
Univariate analysis shows altered taxa in the faecal samples of individuals with helminth infection (red bars) compared to uninfected (blue bars) individuals. Statistically significant **A.** genera, **B.** families and **C.** order are presented. * = *p-value*≤ 0.05; ** = *p-value*≤ 0.01. Complete lists of ANOVA is presented in S3 Table.

Analyses of faecal based on infection status was conducted. Both multivariate and diversity analyses did not show any statistically significant differences in the faecal samples based on infection status (infected/uninfected), however ANOVA did demonstrate specific bacterial species, genera, families and orders that different in abundance between infected and uninfected faecal samples. Complete ANOVA data are presented in **S3 Table**. Reductions in taxa were associated with active helminth infections (**Fig 4**), however genera *Desulfovibrio* (*p-value* = 0.039) and *Oxalobacter* (*p-value* = 0.022) and the family Oxalobacteraceae (*p-value* = 0.022) were instead elevated (**Fig 4A and 4B**).

In this study, analysis of the faecal microbiota demonstrated a reduction in *Haemophilus* in helminth infected people. *Haemophilus parainfluenzae* is normally considered an opportunistic pathogen associated with pulmonary disease [33], but is also reported to be present in faecal samples [34], and is a candidate pathogen for gastritis [35]. In contrast to our findings in the faecal microbiota, *Haemophilus parainfluenzae* has been reported to increase in the bile of individuals harbouring in *Opisthorchis felineus* [36]. Increases shown here in relation to *Desulfovibrio* have also been previously associated with *Ascaris* or *Trichuris* infections in animal models [37] and in clinical observations [14].

### Helminth infections are associated with increased bacterial diversity in saliva microbiota

**Fig 5:**
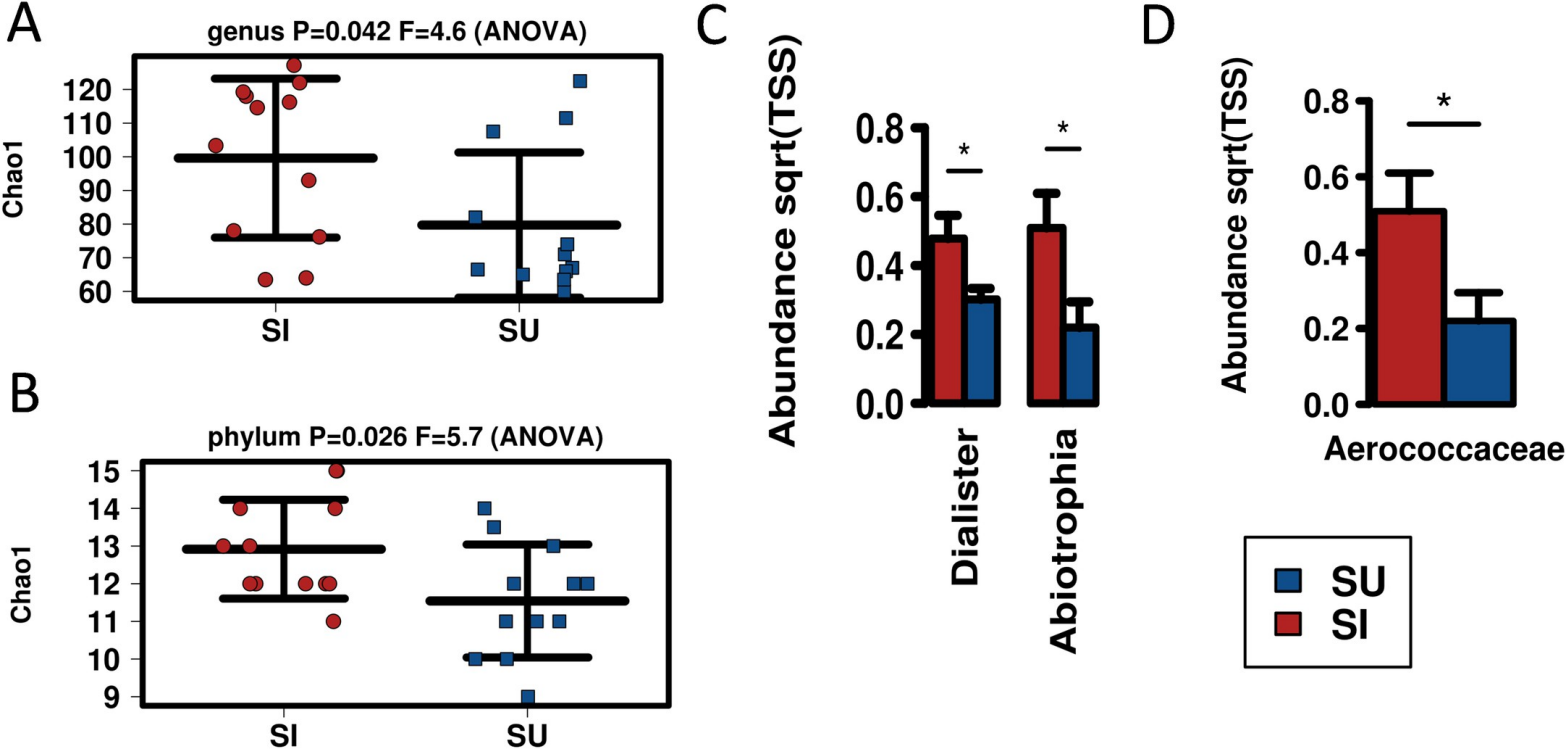
Analysis of saliva samples from infected (red bars) and uninfected (blue bars) participants shows altered microbiota. Chao1 metric indicated elevated alpha diversity associated with infected samples at the **A.** genus and **B.** phylum taxa levels. ANOVA identified **C.** genera, **D.** family all elevated in infected samples. * = *p-value*≤ 0.05. Complete ANOVA data are presented in S4 Table.

Examination of the microbial populations of saliva samples by infection status indicated no statistical differences by multivariate analysis. However, in regard to bacterial alpha diversity, the Chao 1 metric was divergent at both the genus (*p-value* = 0.042) and phylum (*p-value* = 0.026) level, where in both taxonomic ranks, increased alpha diversity was associated with an active helminth infection (SI- saliva infected) (**Fig 5**). Furthermore, ANOVA analysis demonstrated differential bacterial abundance in saliva from infected participants at the specific genera and family level. Complete ANOVA data are presented in **S4 Table**. Two bacterial genera also showed elevated abundance; *Dialister* (*p-value* = 0.027) and *Abiotrophia* (*p-value* = 0.031).

Pathogen infection has also been shown to cause significant changes in the microbial composition of saliva. A recent study of patients infected by the bacterium *Helicobacter pylori* were revealed to have a significantly different microbial composition at the genus level than uninfected subjects, although there was no observed impact to overall diversity [38]. Prevalent infections of *H*. *pylori* are associated with gastric ulcers and gastric cancer [39]. Species that were found to be reduced in abundance during *H*. *pylori* infection included *Aggregatibacter*, *Klebisiella*, *Fusobacterium* and *Parvimonas*, which are also species associated with inflammatory diseases. It was also found that the effect of microbial species upregulation or downregulation during infection was exacerbated upon eradication. The study also inferred some theories on how the oral microbial community may be related to other microbiota on a systemic level through pH changes initiated by pathogen infection. In reviewing the effect of other pathogens on salivary microbial composition it seems likely that helminth infection would also have a significant effect. Currently there is no published research detailing the effect of helminth infections on the oral microbiota.

In our study, univariate analysis of saliva demonstrated a general increase in the abundance of a number of microbes associated with helminth infections. This was also reflected in an increase in the alpha diversity metric, Chao 1, reflecting increased richness. The genus *Dialister* as anaerobic, non-motile and Gram-negative bacilli [40] were elevated. *Dialister* previously been linked to the saliva microbiota and is a known indicator of oral health [41]. *Dialister* is also an oral microbe that has been negatively associated with human diseases including colorectal cancer [42]. In this study the increased levels of saliva *Dialister* may represent a positive indicator of gut health. Links to helminth infections have not been made with these taxa, and certainly not within the saliva microbiota.

### Microbial changes are associated with *Opisthorchis viverrini* infections

Due to the high incidence of *O*. *viverrini* infections in the study region, the microbiota of participants infected with this helminth was analysed independently of other helminth infections. Other helminths species were not considered separately since their presence as a mono-infection were much rarer, compared to *O*. *viverrini*.

**Fig 6:**
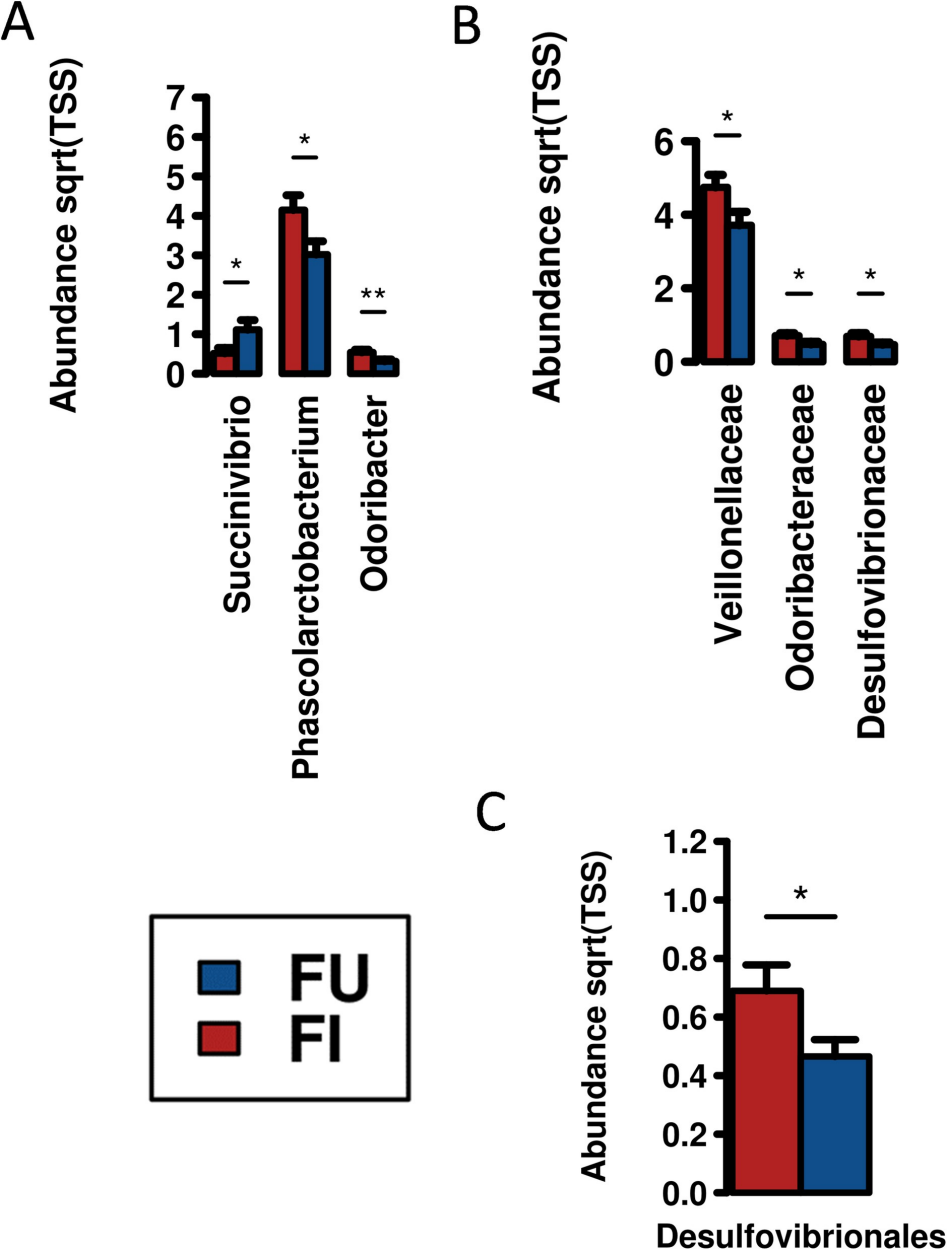
Univariate analysis of faecal samples from *O*. *viverrini* infected (red bars) individuals presented altered microbiota indicators compared to samples from uninfected (blue bars) individuals. Statistically significant **A.** genera, **B.** families and **C.** order are presented. * = *p-value*≤ 0.05; ** = *p-value*≤ 0.01. Complete lists of ANOVA are presented in S5 Table.

*Opisthorchis viverrini* infections either as a single or dual infection (18 samples) were compared to samples from uninfected participants (22 samples) or to other poly-infections where *O*. *viverrini* was absent (26 samples). Compared to uninfected samples *O*. *viverrini* infections resulted in both increases and decreases in specific gut bacterial taxa as shown by univariate analysis (**Fig 6**). Altered genera included *Odoribacter* (*p-value* = 0.0066), *Phascolarctobacterium* (*p-value* = 0.031) and *Succinivibrio* (*p-value* = 0.05). At both the family and order level bacterial taxa were increased in *O*. *viverrini* infections compared to uninfected individuals. Complete lists of ANOVA are presented in **S5 Table**. No statistical differences were noted multivariate analysis or any of the diversity metrics used.

Changes in the saliva microbiota specific to *O*. *viverrini* infections compared to controls were much lower compared to faecal samples mentioned above. Similarly no statistical differences were noted in the multivariate analysis or any of the diversity metrics used. Elevated genera included *Abiotrophia* (*p-value* = 0.028, 2.45 fold), *Unclassified*.*BD15* (*p-value* = 0.034, 2.95 fold) and *Cardiobacterium* (*p-value* = 0.045, 1.63 fold). Elevated families Aerococcaceae (*p-value* = 0.028, 2.45 fold) and Unclassified.BD15 (*p-value* = 0.034, 2.95 fold). The only elevated order was Unclassified.BD15 (*p-value* = 0.034, 2.95 fold), and only elevated phylum was GN02 (*p-value* = 0.034, 2.95 fold).

Next “other” non- *O*. *viverrini* helminth infections were compared to *O*. *viverrini* only infections. Other helminths referred to solo or dual infections excluding *O*. *viverrini*, and consisted primarily of hookworm (*Ancylostoma* spp. and *Necator americanus*), *Ascaris lumbricoides*, *Taenia saginata* and *Trichuris trichiura*. As shown in **S2 Fig**. multivariate analysis presented a clear separation of *O*. *viverrini* and non- *O*. *viverrini* helminth infections (RDA *p-value* = 0.003; CCA *p-value* = 0.001). ANOVA identified species, genera and phyla that were altered, with both elevated and decrease abundance associated with the presence of *O*. *viverrini* compared to other helminth infections. The greatest number of species and genera identified as modulated in faecal samples was demonstrated in this comparison. Complete lists of ANOVA data are presented in **S6 Table**. Eighteen genera including *Haemophilus* (*p-value* = 0.00034), *Sutterella* (*p-value* = 0.0004), *Phascolarctobacterium* (*p-value* = 0.0008), *Odoribacter* (*p-value* = 0.0019) and *Megamonas* (*p-value* = 0.0038) were statistically different in abundance.

The saliva microbiota was not analysed to compare non- *O*. *viverrini* helminth infections to *O*. *viverrini* only infections, since only 2 of the 12 saliva samples that were positive for helminths were not *O*. *viverrini* co-infected.

*Opisthorchis viverrini* infections were considered a separately due to the prominence of the helminth in the region. As a fish borne trematode, *O*. *viverrini* is endemic in areas of South East Asia particularly Thailand where an estimated 8 million people are infected and is acquired when it is ingested from eating raw or undercooked fish [43,44]. Opisthorchiasis has been shown to be a definitive cause of human cancer by the World Health Organisation’s International Agency Research on Cancer [45]. In comparing *O*. *viverrini* infections and mixed helminth infections, both increases and decreases in taxa were identified. In a report using the nematode *Haemonchus contortus*, an altered gut microbiota was seen in the goat host, including increases of several *Veillonellaceae* [46], this mirrors what observations from clinical samples in this study. Plieskatt and colleagues [44], discovered that infections in an animal model for *O*. *viverrini* resulted in the increased numbers of *Lachnospiraceae*, *Lactobacillaceae* and *Ruminococcaceae* whilst the levels of *Eubacteriaceae*, *Porphyromonadaceae* and *Erysipelotrichaceae*, and all decreased. In their study [44] they noted the presence of *Desulfovibrionaceae* but did not see any change in abundance due to the presence of infection, unlike in our study where increases were noted.

## Conclusions

The 16S rRNA microbiota analysis we present here has limitations in the information that can be gained. A more complete understanding of the taxa presented would need more extensive and more expensive approaches. This is particularly the case for our data at the species taxa rank which are “unclassified”, would benefit from a more comprehensive approach, such as metagenomics [47]. As such we have limited our presentation of data and discussion to genera or higher taxonomic ranks.

Through reviewing the information currently available on helminth modulation of microbiota structure, it is clear that there are still gaps in our knowledge, especially concerning microbiota alterations on a systemic level. Much research is available on the effect of pathogen infection on the gut and faecal microbiota yet there is currently no research available concerning the impact of helminth infection on the saliva microbiota. Oral microbiota research is important for both its connection to systemic diseases and its potential for unlocking new diagnostic methods. The human gut microbiota is inhabited by an array of microbes with bacteria a major component. In many countries the presence of helminth parasites greatly impacts on the health and the quality of life of people; of which disruption of the microbiota is an integral element. Many bacteria exert a variety of influences on the human gut and can be considered positive or detrimental to human health [48]. The human gut microbiota has received increasing attention especially in recent years [48]. High-throughput technologies such as next generation sequencing have allowed for a deep insight into the composition of the gut microbiota as well as its functions. Even though there has been an array of studies conducted on this topic, many questions still remain unanswered [49]. Age, genetics, diet, and parasitic infections are just a few of these factors that can lead to both moderate and drastic alterations of the gut microbiota composition and abundance [50].

In the future a larger dataset would enable the comparison of more parameters across individuals, beyond the presence of helminths or more specifically to the region, the presence of *O*. *viverrini*. As presented in our first study [6] regional differences occur across Thailand in the helminth species present. This variable could be considered further with access to a larger cohort. Other important factors that could influence an individual’s microbiome include socio-economic status, profession, diet and genetic markers. These factors could be considered in conjunction with an active helminth infection to better understand host microbiota changes. It is hoped these results here will encourage larger studies to consider faecal and oral microbiota and microbiomes associated with neglected tropical diseases.

Participants in the original study were recruited in a village rather than clinic/hospital setting. Thus all were not presenting overt symptoms at the time of tissue collection. The chronic nature of many of the helminth morbidities present make the identification of symptomatic individuals difficult, the collection of further clinical parameters may be useful in the future. The linking of microbiota changes with the presentation of symptoms related to helminthiases would be a useful future goal of this type of research. Larger studies if undertake could stratify both the presentations of symptoms and associated infection intensities. This would be very useful in order to draw conclusions across those parameters in relation to any microbial changes.

The human gut not only houses the normal microbiota flora it also provides an environment to parasites including helminth species. Many recent studies have supported the hypothesis that infections caused by helminth species impact, directly or indirectly on the composition of the human gut microbiota composition [51]. Despite the ever-growing literature dissecting the interactions between helminth infections and the gut microbiota, this relationship is still poorly understood. It is hypothesised that when a helminth establishes infection it alters the gut microbiota by both excretory and secretory molecules [52].

Through previous murine model studies and some human studies, it has been revealed that helminth infection has a significant impact on the composition and colonization potential of intestinal gut microbiota [16]. Much interest has developed from these studies in the potential of adapting helminth derived molecules (ES or excreted/secreted) as therapeutics to reduce both inflammatory and fibrotic diseases [53–55]. There has also been interest generated in observing and tracking microbiota shifts during infection states as a means to develop a new diagnostic method for parasitic helminths [56]. A new diagnostic test that tracks significant shifts in oral microbiota associated with helminth infection would be very advantageous. In such an assay sample collection would be much more convenient and would not require staff that are highly trained in microscopy. However, compared to the much lower costs associated with microscopy, routine microbiota would not be feasible in the near future in most low to middle income countries associated with endemic helminthiases. These novel observations of the impact of helminths have on the human microbiota will provide a more complete view of parasitism.

## Supporting information

S1 File Biome format file. (BIOM)

S1 Table Metadata file containing- Sample ID, Label, Individual, Infection category, Province, Sex, Age, Code ID, Infection by PCR or KK, Groups by PCR or KK, Parasite species richness (PCR or KK), Faecal or Saliva, FS infection and number, Faecal Saliva matched and Ov present. (XLSX)

S2 Table ANOVA analysis of the four sample types. Results are stratified (separate tabs) at the OTU, species, genus, family, order, and phylum levels. Results include *p- value* based in Infection category, False Discover Rate (FDR), adjusted *p- value* based on Tissue and Infection status, Paired *p- value* (Tukey), and mean abundance for each Tissue and Infection condition.
(XLSX)

S3 Table ANOVA analysis of faecal samples by infection status only. Results are stratified (separate tabs) at the species, genus, family, and order levels. Results include *p- value* based in Infection category, False Discover Rate (FDR), adjusted *p- value* based on Tissue and Infection status, Paired *p- value* (Tukey), and mean abundance for each Tissue and Infection condition.
(XLSX)

S4 Table ANOVA analysis of saliva samples by infection status only. Results are stratified (separate tabs) at the species, genus, and family levels. Results include *p- value* based in Infection category, False Discover Rate (FDR), adjusted *p- value* based on Tissue and Infection status, Paired *p- value* (Tukey), and mean abundance for each Tissue and Infection condition.
(XLSX)

S5 Table ANOVA analysis of faecal samples by presence of *O. viverrini* infection status compared to uninfected individuals. Results are stratified (separate tabs) at the species, genus and family levels. Results include *p- value* based on Infection category, False Discover Rate (FDR), adjusted *p- value* based in Tissue and Infection status, Paired *p- value* (Tukey), and mean abundance for each Tissue and Infection condition.
(XLSX)

S6 Table ANOVA analysis of faecal samples by presence of *O. viverrini* infection status compared to other helminth infected individuals. Results are stratified (separate tabs) at the species, genus and phylum levels. Results include *p- value* based in Ov present category, False Discover Rate (FDR), adjusted *p- value* based on Tissue and Infection status, Paired *p- value* (Tukey), and mean abundance for each Tissue and Infection condition.
(XLSX)

S1 Fig Indicators of sequencing quality coloured by tissue type and infection status. **A.** Rarefaction of individual samples. **B.** Reads per sample including Kitome control. **C.** Box plot of read per sample grouped by sample type. **D.** Boxplot of read per sample grouped by sample type. Excluding Kitome control. ANOVA of boxplot by tissue and infection status presented a difference in reads (*P-value* = 0.02329). FU = Faecal Uninfected, FI = Faecal Infected, SU = Saliva Uninfected, SI = Saliva Infected, K = Kitome.
(TIF)

S2 Fig Multivariate and univariate analysis of faecal sample of helminth infected individuals. Infections with (blue) or without (red) the presence of *O*. *viverrini* (single or dual) were compared to other helminth diseases. Multivariate analysis was performed at the OTU level. **A.** Unsupervised Principal Coordinates Analysis (PCoA) **B.** Redundancy Analysis (RDA) and **C.** Canonical Correspondence Analysis (CCA) both demonstrated differences between *O*. *viverrini* and non- *O*. *viverrini* infections that were statistically significant (RDA *p-value* = 0.003; CCA *p-value* = 0.001). Univariate analysis by ANOVA and statistically significant **D.** genera, **E.** phylum are presented. * = *p-value*≤ 0.05; ** = *p-value*≤0.01. *** = *p-value*≤0.001. Complete lists of ANOVA are presented in S6 Table.
(TIF)
