# Supplementary figures and images for: Clinical helminth infections alter host gut and saliva microbiota
Source: PLoS Negl Trop Dis. 2022 Jun 8;16(6):e0010491. doi: 10.1371/journal.pntd.0010491 (PMC9212162; doi:10.1371/journal.pntd.0010491)

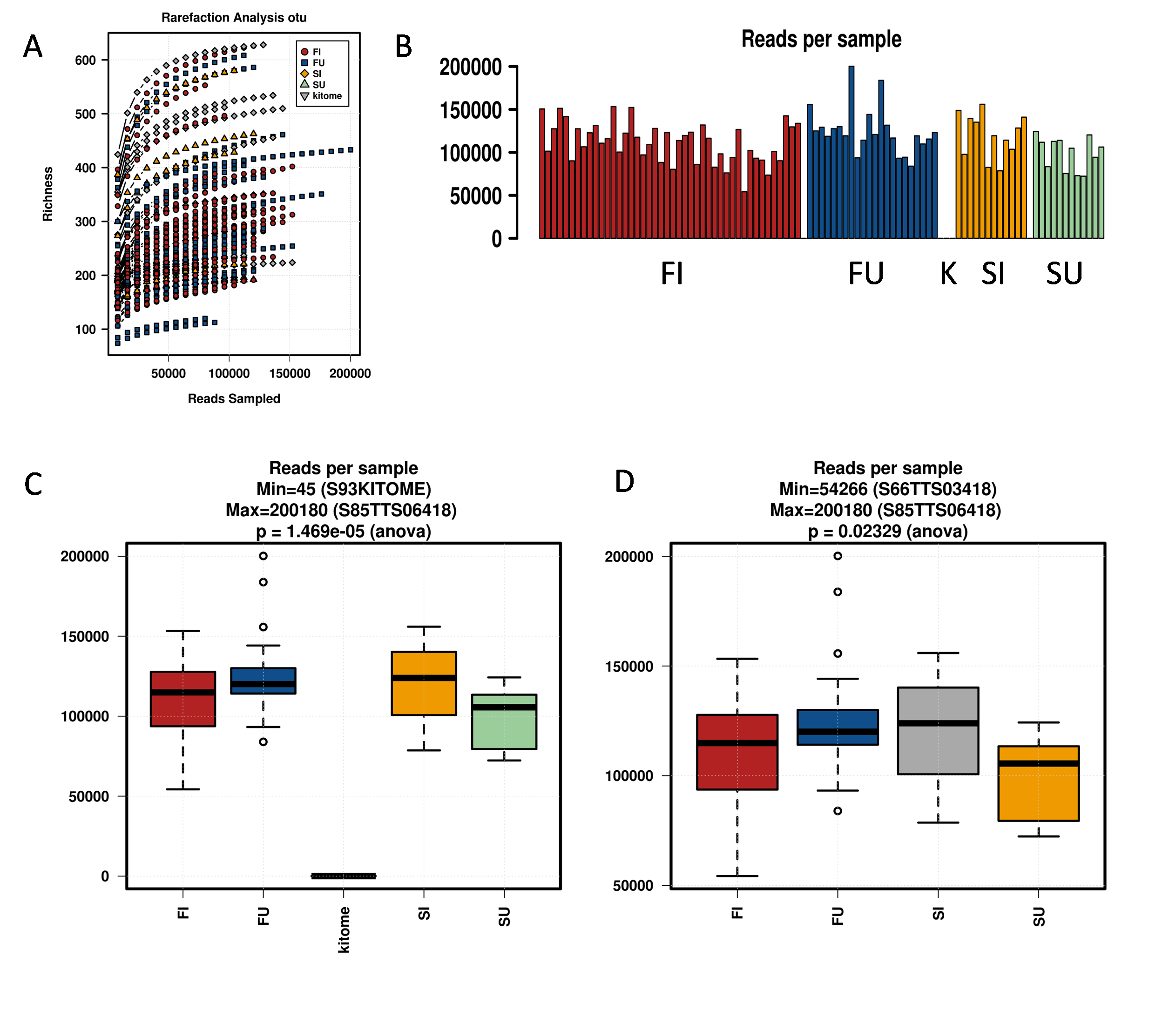

Supplement: S1 Fig — A. Rarefaction of individual samples. B. Reads per sample including Kitome control. C. Box plot of read per sample grouped by sample type. D. Boxplot of read per sample grouped by sample type. Excluding Kitome control. ANOVA of boxplot by tissue and infection status presented a difference in reads (P-value = 0.02329). FU = Faecal Uninfected, FI = Faecal Infected, SU = Saliva Uninfected, SI = Saliva Infected, K = Kitome. (TIF) [file pntd.0010491.s008.tif]

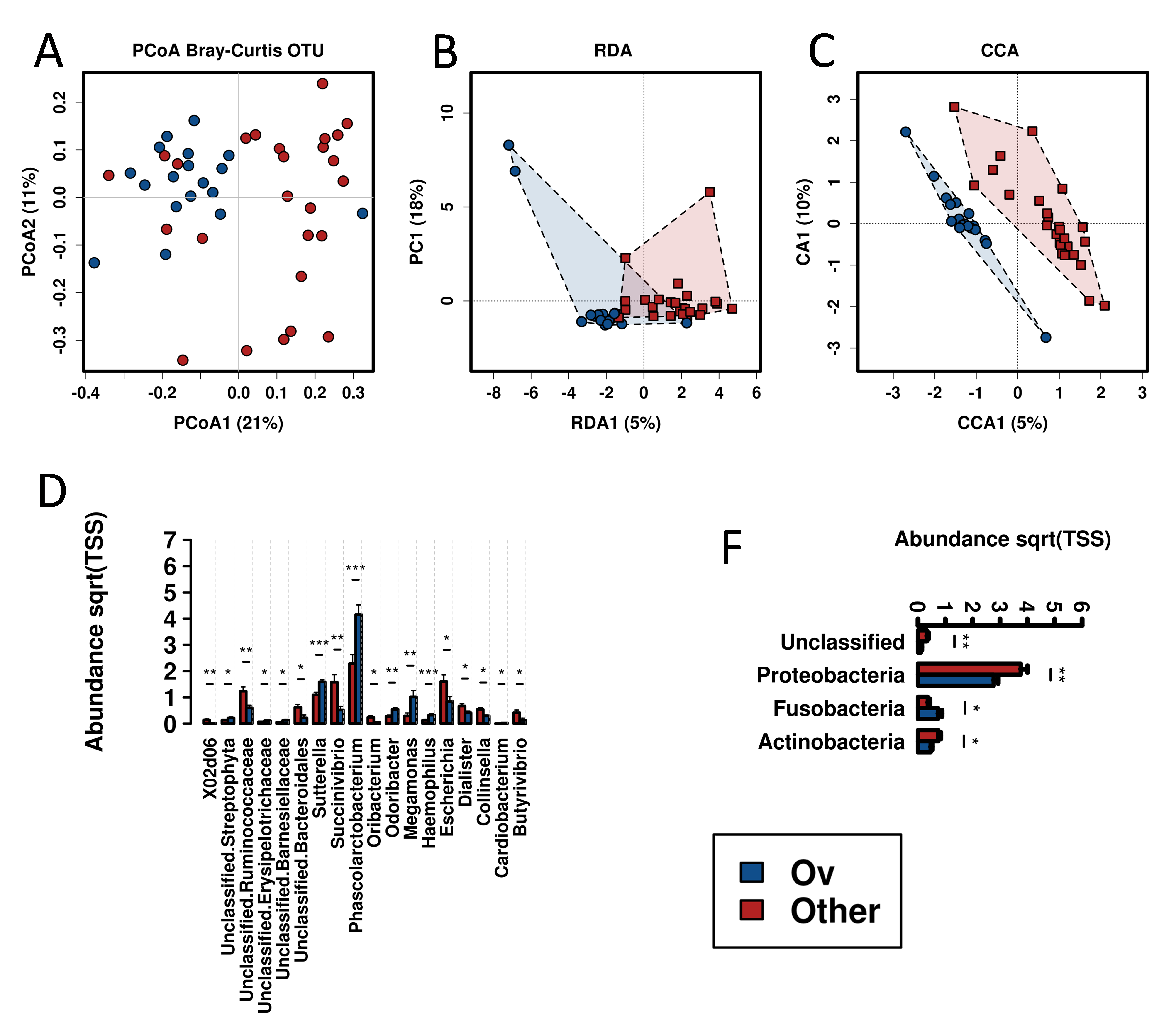

Supplement: S2 Fig — Infections with (blue) or without (red) the presence of O. viverrini (single or dual) were compared to other helminth diseases. Multivariate analysis was performed at the OTU level. A. Unsupervised Principal Coordinates Analysis (PCoA) B. Redundancy Analysis (RDA) and C. Canonical Correspondence Analysis (CCA) both demonstrated differences between O. viverrini and non- O. viverrini infections that were statistically significant (RDA p-value = 0.003; CCA p-value = 0.001). Univariate analysis by ANOVA and statistically significant D. genera, E. phylum are presented. * = p-value≤ 0.05; ** = p-value≤0.01. *** = p-value≤0.001. Complete lists of ANOVA are presented in S6 Table. (TIF) [file pntd.0010491.s009.tif]
